# microRNA-29b prevents liver fibrosis by attenuating hepatic stellate cell activation and inducing apoptosis through targeting PI3K/AKT pathway

**DOI:** 10.18632/oncotarget.2621

**Published:** 2014-10-22

**Authors:** Jia Wang, Eagle S.H. Chu, Hai-Yong Chen, Kwan Man, Minnie Y.Y. Go, Xiao Ru Huang, Hui Yao Lan, Joseph J.Y. Sung, Jun Yu

**Affiliations:** ^1^ Institute of Digestive Disease and The Department of Medicine and Therapeutics, State Key Laboratory of Digestive Disease, Li Ka Shing Institute of Health Sciences, The Chinese University of Hong Kong, Hong Kong; ^2^ Gastrointestinal Cancer Biology & Therapeutics Laboratory, CUHK-Shenzhen Research Institute, Shenzhen, China; ^3^ Department of Surgery, LKS Faculty of Medicine, The University of Hong Kong, Hong Kong

**Keywords:** miR-29b, hepatic stellate cell, liver fibrosis, AKT3

## Abstract

microRNA-29b (miR-29b) is known to be associated with TGF-β-mediated fibrosis, but the mechanistic action of miR-29b in liver fibrosis remains unclear and is warranted for investigation. We found that miR-29b was significantly downregulated in human and mice fibrotic liver tissues and in primary activated HSCs. miR-29b downregulation was directly mediated by Smad3 through binding to the promoter of miR-29b in hepatic stellate cell (HSC) line LX1, whilst miR-29b could in turn suppress Smad3 expression. miR-29b transduction in the liver of mice prevented CCl_4_ induced-fibrogenesis, concomitant with decreased expression of α-SMA, collagen I and TIMP-1. Ectopic expression of miR-29b in activated HSCs (LX-1, HSC-T6) inhibited cell viability and colony formation, and caused cell cycle arrest in G1 phase by downregulating cyclin D1 and p21^cip1^. Further, miR-29b induced apoptosis in HSCs mediated by caspase-9 and PARP. miR-29b inhibited its downstream effectors of PIK3R1 and AKT3 through direct targeting their 3′UTR regions. Moreover, knockdown of PIK3R1 or AKT3 suppressed α-SMA and collagen I and induced apoptosis in both HSCs and in mice. In conclusion, miR-29b prevents liver fibrogenesis by inhibiting HSC activation and inducing HSC apoptosis through inhibiting PI3K/AKT pathway. These results provide novel mechanistic insights for the anti-fibrotic effect of miR-29b.

## INTRODUCTION

Hepatic fibrosis is an integral part in the progression of chronic inflammatory liver disease featured with the excessive accumulation of extracellular matrix (ECM) proteins. With prolonged liver damage, fibrosis may progress to cirrhosis and primary liver cancer [1]. Unlike the irreversible cirrhosis, hepatic fibrosis is a reversible disease, and an effective treatment can be able to prevent or reverse the fibrotic process [2]. Hepatic stellate cells (HSCs) play a key role in liver fibrogenesis [3]. HSCs are quiescent in normal liver but will be activated in response to liver damage [4]. Activated HSCs, on one side, secrete transforming growth factor beta-1 (TGF- β1), the most potent fibrogenetic factor, to induce collagen production and ECM accumulation [5,6]; on the other side, activated HSCs inhibit the activation of matrix metalloproteinases (MMPs) by up-regulation of tissue inhibitors of metalloproteinases (TIMPs), and cause reduction of matrix degradation [7,8]. This imbalance between ECM degradation and accumulation results in occurrence of liver fibrosis. Therefore, controlling the activation of HSCs is considered as a promising therapy to antagonize liver fibrosis. There is a decrease in the number of activated HSCs when fibrosis resolves. The fate of the activated HSCs is either returning to a quiescent state, or undergoing apoptosis [9]. There is pressing need for effective molecular targeted therapies for liver fibrosis through regulating HSCs activation.

microRNAs (miRNAs) are endogenous small non-coding RNAs that control gene expression by degrading target mRNA or suppressing their translation at the 3′-untranslated regions (3′-UTR). miRNAs play fundamental roles in a variety of cellular processes including cell differentiation and proliferation [10]. Aberrant expression of miRNA is reported to be associated with the liver diseases including viral hepatitis [11], fatty liver disease [12] and liver cancer [13]. It is reported recently that miRNAs can regulate the activation of HSCs and thereby regulate liver fibrosis [14]. Identification of key abnormally expressed miRNA in pathologic state will be helpful to further understand the disease mechanism, and modulation of its activity may be of therapeutic benefit. We have recently reported that miR-29b, a negative regulator for the Smad3 and type I collagen is a key regulator in renal fibrosis [15] and pulmonary fibrosis [16]. However, the biological role of miR-29b in liver fibrosis and its possible contribution acting as a protective factor against liver fibrogenesis remain largely unclear. In this study, we first determined whether aberrant expression of miR-29b exists in liver fibrosis, and found that miR-29b was significantly downregulated in fibrotic liver tissues from human and rodent model, and in activated HSCs. The functional role and therapeutic potential for miR-29b in liver fibrogenesis were therefore characterized *in vivo* using an ultrasound-microbubble-mediated miR-29b transfer, and *in vitro* by overexpression of miR-29b in HSCs. We revealed that miR-29b prevents hepatic fibrogenesis in mice by attenuating HSCs activation and inducing HSCs apoptosis. In particular, we demonstrated by a variety of *in vitro* and *in vivo* approaches that phosphoinositide-3-kinase regulatory subunit 1 (PIK3R1) and protein kinase B (AKT3) are direct targets of miR-29b in HSCs responsible for signaling onset of HSCs activation and liver fibrosis.

## RESULTS

### miR-29b is down-regulated in human fibrotic liver tissues

**Figure 1:**
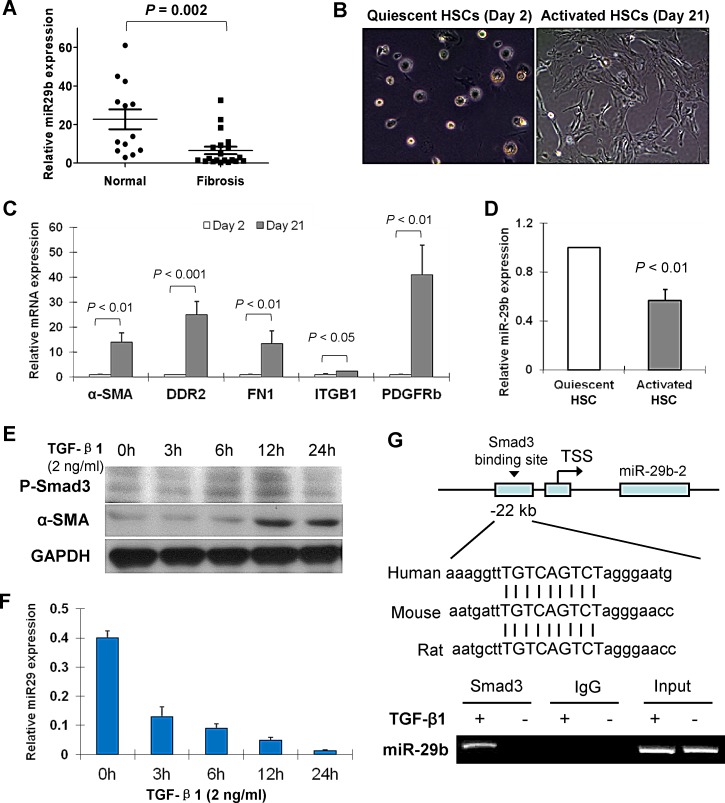
miR-29b is downregulated in liver fibrosis and in activated hepatic stellate cells (HSCs) and down-regulation of miR-29b is mediated by Smad3 (A) Level of miR-29b was significantly lower in human fibrotic liver tissues (n = 20) than in normal liver tissues (n = 13). (B) Representative morphological images of primary cultured quiescent HSCs and activated HSCs of rat origin. (C) mRNA expression of key genes involved in the activation of HSCs including α-SMA, DDR2, FN1, ITGB1 and PDGFRb were analyzed by real-time PCR. (D) miR-29b expression is reduced in activated HSCs compred to the quiescent HSCs. (E) Human HSC cell line LX1 was treated with TGF-β1 (2 ng/ml) for 0, 3h, 6h, 12h and 24h, respectively. Protein expression of phosphorylated-Smad3 (p-Smad3) and α-SMA was determined by Westerb blot, GAPDH was used as loading control. (F) miR-29b expression was examined by real-time RT-PCR. (G) DNA sequence alignments indicate a highly conserved Smad3 binding site at the promoter region 22kb upstream of miR-29b in difference species (upper panel). Functional interaction between Smad3 and miR-29b promoter was evaluated by Chromatin Immunoprecipitation (ChIP)-PCR. LX1 cells were treated with TGF-β1 (2ng/ml) for 24 h, then cross-linked with formaldehyde and lysed. The soluble chromatin was immunoprecipitated with the anti-Smad3 Ab. Two pairs of primers were designed to detect the Smad3-containing promoter region of miR-29b by ChIP-PCR (lower panel) and a direct interaction be Smad3 and miR-29b was demonstrated.

We first assessed the expression of miR-29b in 20 human liver fibrosis tissues and 13 normal human liver biopsies. As determined by real-time PCR, miR-29b expression level was significantly lower in fibrotic tissues comparing to the normal liver tissues (*P* = 0.002) (Figure 1A).

### miR-29b is expressed in primary quiescent HSCs, but down-regulated in activated HSCs

Freshly isolated rat HSCs retained their quiescent phenotype with distinct stellate morphology when cultured on plastic for 1-2 days (Figure 1B). They became fully activated after cultured for more than 7 days with the typical cell morphology of a large, spread out and flattened polygonal shape observed at day 21 day (Figure 1B). mRNA expression of the key genes involved in the activation of HSCs including α-SMA, discoidin domain receptor 2 (DDR2), fibronectin 1 (FN1), integrin â1 (ITGB1) and platelet-derived growth factor receptor-β (PDGFR-β) was up-regulated in day 21 HSCs as compared to day 2 HSCs (Figure 1C), confirming the activation status of day 21 HSCs from the primary culture. We found that miR-29b was abundant in quiescent HSCs, but was significantly decreased in activated HSCs (Figure 1D).

### Down-regulation of miR-29b is mediated by Smad3

We have previously reported that miR-29b is a downstream target gene of Smad3 and it is negatively regulated by TGF-β/Smad signaling in renal fibrosis [15]. We evaluated whether the downregulation of miR-29b in liver fibrosis was mediated by Smad3 and their potential interaction. Human HSC cell line LX1 was treated with TGF-β1 (2 ng/ml) for 0, 3h, 6h, 12h and 24h, respectively. Protein expression of phosphorylated-Smad3 (p-Smad3) and α-SMA was increased in a time dependent manner (Figure 1E). However, the miR-29b expression showed a time-dependent decrease by TGF-β1 in LX1 cells (Figure 1F), indicating that miR-29b is a potential downstream target of TGF-β/Smad3 signaling.

We further determined the interaction between Smad3 and miR-29b. We have previously reported that a Smad response element (TGTCAGTCT) is located at ~22kb upstream of miR-29b, a highly conserved region [15]. By chromatin immunoprecipitation (ChIP)-PCR assay, we revealed the directly binding of Smad3 to miR-29b promoter in LX1 cells with TGF-β1 treatment (Figure 1G), indicating that miR-29b is a direct transcriptional target of Smad3 in HSCs.

### miR-29b prevents carbon tetrachloride (CCl4)-induced liver fibrosis in mice

**Figure 2:**
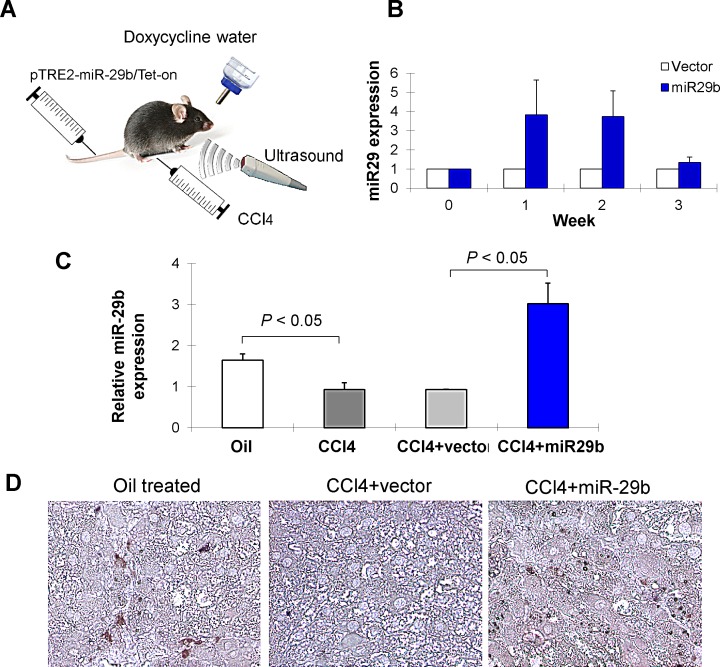
Ultrasound-mediated gene transfer of miR-29b in CCl-induced liver fibrosis in C57BL6 mice (A) C57/BL6 mice were administered CCl_4_ twice per week by Intraperitoneal injection for 8 weeks to induce liver fibrosis. Separately, CCl_4_ treated mice were introduced with miR-29b using pTRE2-miR-29b-Tet-on plasmid or control vector by tail vein injection, followed by 5 min ultrasound treatment (2W/cm2) transcutaneously on liver location as described in Materials and Methods. (B) The efficacy of miR-29b delivery to the liver of mice was determined by quantitative real-time PCR and consistent miR-29b expression was detected at week 1 and week 2. Thus, a tail vein injection was given for 3 times in 8 weeks duration in mice treated with CCl_4_. (C) Over-expression of miR-29b was confirmed in miR-29b-transfected mice in the liver by real-time RT-PCR and (D) by *in situ* hybridization.

We next examined whether introduction of miR-29b by gene transfer could ameliorate CCl_4_-induced liver fibrosis *in vivo*. The Doxycycline-inducible miR-29b expressing plasmids (pTRE2-miR-29b/pTet-on) were transfected into the liver through tail vein injection followed by ultrasound treatment transcutaneously at the liver location (Figure 2A). In our pilot study, the efficacy of miR-29b delivery to the liver in mice was detected by real-time PCR and consistent miR-29b expression was detected at week 1 and week 2 (Figure 2B). In this connection, a tail vein injection was given for 3 times in 8 weeks duration in mice treated with CCl_4_. Over-expression of miR-29b was confirmed in miR-29b-transfected mice in the liver by real-time RT-PCR (Figure 2C) and by miR-29b *in situ* hybridization (Figure 2D).

**Figure 3:**
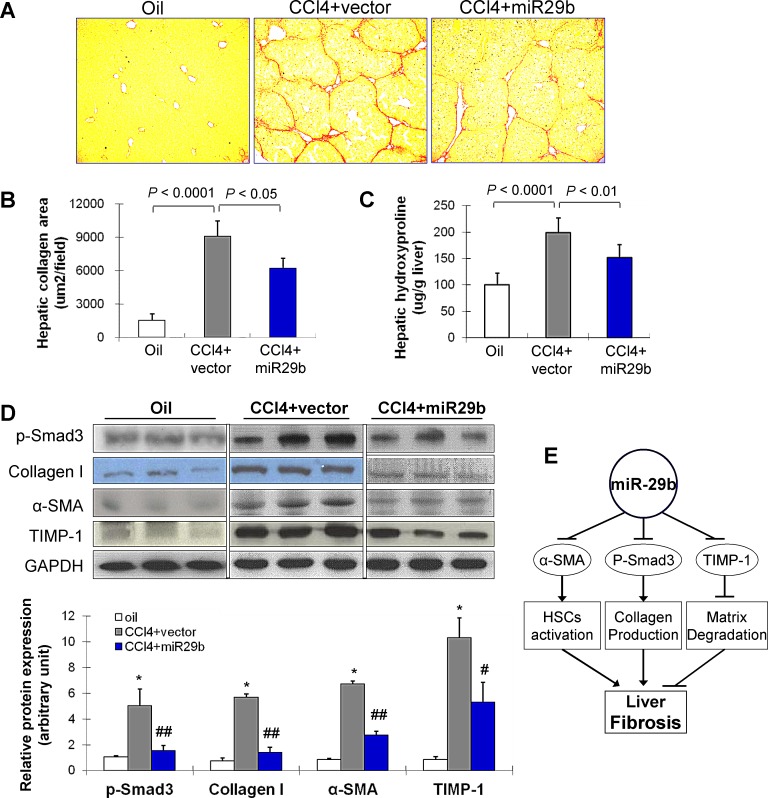
Gene transfer of miR-29b prevents CCl4-induced liver fibrosis in mice (A) Collagen deposition by Picrosirius red staining of liver. (B) Semi-quantitative analysis of collagen area by Picrosirius red staining. (C) The quantification of collagen in liver tissues of mice was displayed by content of hydroxyproline (ug/mg liver) in different treatment groups. (D) Effects of CCl_4_ and transduced with miR-29b on hepatic protein expression of p-Smad3, collagen I, α-SMA and TIMP-1 by Western blot. GAPDH was used as loading control. Values are mean ± SD, *P < 0.001 compared with control mice (treated with oil); #*P* < 0.01, ##*P* < 0.001 compared with mice treated with CCl_4_. (E) Proposed scheme of the mechanism by which miR-29b prevents liver fibrogenesis in mice.

As shown in Figure 3A, significant bridging fibrosis, fibrous septa and cirrhotic nodules were observed in liver sections in mice treated with CCl_4_ and transduced with control vector for 8 weeks by Picrosirius red-staining; whilst, transduction of miR-29b caused marked reduction in the distribution of collagen fibers. Morphometric analysis yielded concordant results where the Picrosirius red-stained collagen areas were significantly reduced in miR-29b introduced mice compared to the control vector transfected mice (*P* < 0.05) (Figure 3B). Moreover, quantitation of collagen by measuring the amount of hepatic hydroxyproline content supported the significant improvement of liver fibrosis by miR-29b delivery (*P* < 0.01) (Figure 3C).

### miR-29b suppresses genes involved in fibrogenesis

We examined molecular factors involved in HSC activation (α-SMA), collagen production (p-Smad3, collagen I) and matrix degradation (TIMP-1) by Western blot. Introduction of miR-29b significantly reduced protein expression of p-Smad3, collagen I, α-SMA and TIMP-1 (Figure 3D), inferring that the anti-fibrosis effect of miR-29b was mediated at least by suppressing genes involved in fibrogenesis (Figure 3E).

### miR-29b reduces the activation of hepatic stellate cells *in vitro*

**Figure 4:**
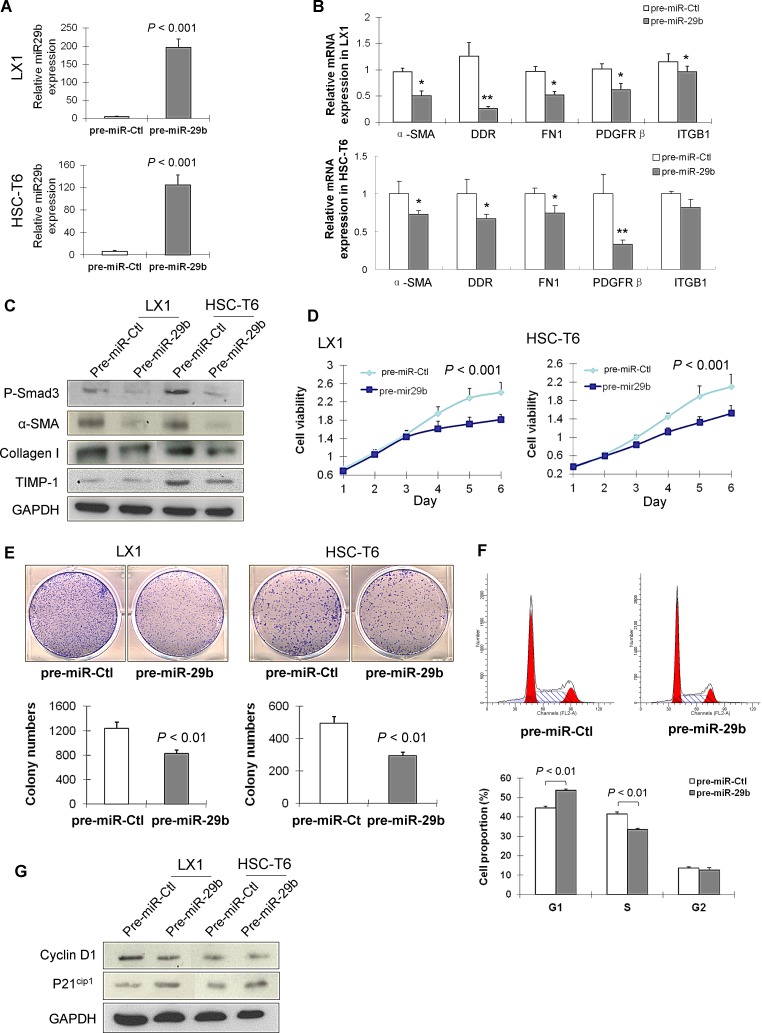
miR-29b inhibits HSC proliferation and arrests cell cycle in G1 phase (A) Ectopic expression of miR-29b in HSC cell lines LX1 and HSC-T6 was confirmed by RT-PCR. (B) mRNA expression of α-SMA, DDR2, FN1, ITGB1 and PDGFRb in miR-29b expressed HSCs and control HSCs by real-time PCR. (C) Eectopic expression of miR-29b suppressed protein expression of p-Smad3, α-SMA, collagen I and TIMP-1 in LX1 and HSC-T6. (D). miR-29b inhibited HSC cell growth as determined by cell viability assay, and (E) by colony formation assay. (F) miR-29b increased G1 phase cell population, but decreased S phase cell population as examined by flow cytometry analysis. (G) Protein expression of key G1 cell cycle regulators cyclinD1 and p21^cip1^ by Western blot. GAPDH was used as loading control.

Given the crucial role of miR-29b in suppressing liver fibrosis *in vivo*, we examined whether miR-29b plays any part in modulation of the activation of HSC *in vitro*. To test this, two activated HSC cell lines (LX-1 and HSC-T6) were transfected with pre-miR-29b or pre-miR-control (Figure 4A). Introduction of miR-29b resulted in significant down-regulation of α-SMA, DDR2, FN1, ITGB1 and PDGFR-β mRNAs (Figure 4B). This was confirmed by the downregulation of protein expression of p-Smad3, α-SMA, collagen I and TIMP-1 (Figure 4C), inferring the suppressed activation of HSCs by miR-29b.

### miR-29b inhibits HSCs proliferation by causing cell cycle arrest in G1 phase

To evaluate the effects of miR-29b on cell proliferation, LX-1 and HSC-T6 cells were transfected with either pre-miR-29b or pre-miR-control. As determined by cell viability assay, miR-29b significantly suppressed cell growth in LX-1 and HSC-T6 cells (*P* < 0.001) (Figure 4D). The growth suppressive effect of miR-29b in HSC cells was further confirmed by a colony formation assay, which showed the colonies formed in LX1 and HSC-T6 cells transfected with miR-29b were significantly less than those of control cells (Figure 4E).

To determine the mechanism by which miR-29b inhibited HSC cells proliferation, we examined the effect of miR-29b on cell cycle distribution. Ectopic expression of miR-29b in LX1 led to a significant increase in the G1 phase population (*P* < 0.01; Figure 4F), and a corresponding reduction in the S phase cells (*P* < 0.01; Figure 4F). Western blot analysis revealed that miR-29b suppressed the G1-S transition promoter cyclin D1 and induced the G1 gatekeeper P21^Cip1^ (Figure 4G), further confirming the effect of miR-29b in blocking the cell cycle at the G1/S checkpoint.

### miR-29b induces apoptosis in HSCs

**Figure 5:**
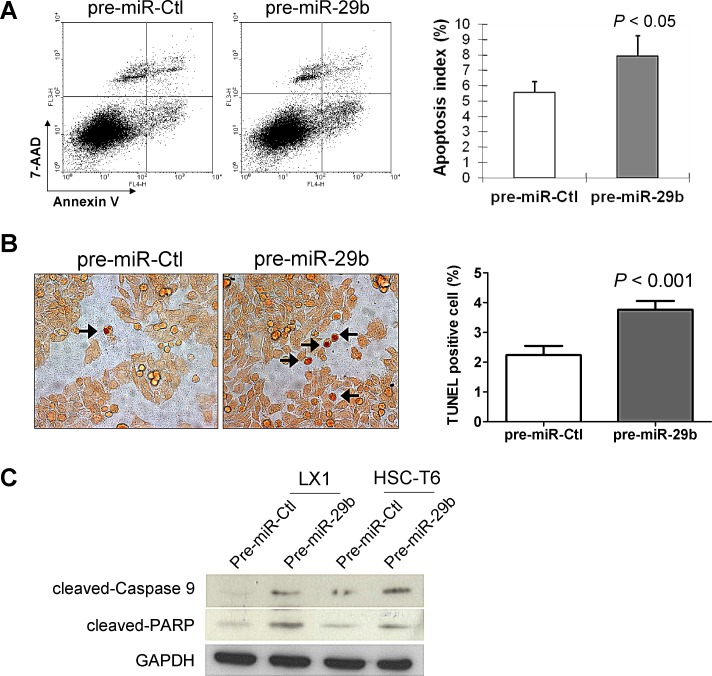
miR-29b induces HSC apoptosis (A) HSCs apoptosis was determined by Annexin V/7-AAD staining and analyzed by flow cytometry. Annexin V positive apoptotic cells were significantly increased in LX1 cells transfected with miR-29b compared with those transfected with control-miR. Data are mean ± SD from four independent experiments in duplicate. (B) Cell apoptosis in HSCs was examined by TUNEL staining. Apoptosis index was quantified by counting the proportion of TUNEL-positive cells. (C) Effects of miR-29b on protein expression of apoptosis-related genes by Western blot. GAPDH was used as loading control.

In order to determine whether the observed suppressive effect of cell growth by miR-29b was due to an induction of apoptosis, cell apoptosis was evaluated by Annexin V/7-AAD double staining and flow cytometry. Ectopic expression of miR-29b in LX1 cells caused a significant increase of apoptotic cells (*P* < 0.05, Figure 5A). Moreover, the apoptotic index quantified by transferase-mediated nick-end labeling (TUNEL) positive cells was significantly increased in LX1 transfected with pre-miR-29b compared with pre-miR-control (*P* < 0.001) (Figure 5B). In keeping with this, protein expression of the active forms of key apoptosis genes including cleaved caspase-9 and cleaved PARP was enhanced in LX-1 cells transfected with pre-miR-29b compared to pre-miR-control by Western blot (Figure 5C). These findings indicate that miR-29b induces cell death and promotes subsequent proliferative activity in HSCs.

### miR-29b inhibits PIK3R1 and AKT3 by direct binding to their 3′-UTR regions

**Figure 6:**
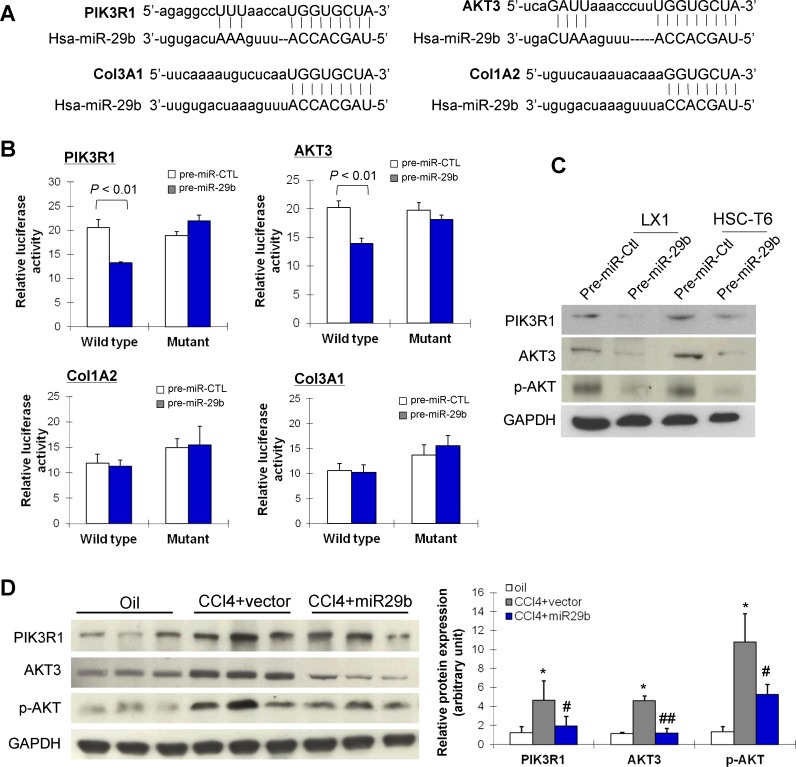
miR-29b inhibits liver fibrosis and suppresses the activation of HSCs through direct targeting PIK3R1 and AKT3 (A) miR-29b potential binding sites on the 3′-UTR of four candidate genes, PIK3R1, AKT3, Col1A2 and Col3A1. (B) LX1 cells were transfected with firefly luciferase transcript containing either wild-type or mutant form of 3′-UTR of the 4 candidate genes, in the presence of either control or miR-29b precursor, and then assessed for luciferase reporter activity at 48 hours post-transfection. The luciferase reporter activity of PIK3R1ang AKT3 was suppressed by wildtype miR-29b. (C) Protein expression of PIK3R1, AKT3 and p-AKT3 was reduced by miR-29b in LX1 and in HSC-T6 cells. (D) PIK3R1, AKT3 and p-AKT expressions which were induced in CCl_4_-treated mice were down-regulated in mice with gene transfer of miR-29b. Values are mean ± SD, *P < 0.01 compared with control mice (treated with oil); #*P* < 0.05, ##*P* < 0.01 compared with mice treated with CCl_4_.

We then searched for the targets of miR-29b by in silico searches using two prediction algorithms miRanda and TargetScan. We found that the 3′-UTR of PIK3R1, AKT3, Col3A1 and Col1A2 contain putative binding sites for miR-29b (Figure 6A). To verify whether miR-29b directly binds to the 3′-UTR of these candidate genes and causes translational inhibition, we constructed pMIR-report plasmids encoding a firefly luciferase transcript with either wild-type or mutant 3′-UTR of PIK3R1, AKT3, Col1A2 and Col3A1 (Figure 6B). We evaluated their respective luciferase reporter activity after co-transfection with pre-miR-29b or pre-miR-control in LX-1 cells. The results showed that, pre-miR-29b repressed the reporter activity of the transcript containing wild-type 3′-UTR of PIK3R1 (*P* < 0.01) and AKT3 (*P* < 0.01), but not Col1A2 and Col3A1, compared with pre-miR-control. Whist miR-133a had no inhibition effect on the reporter activity of the mutant 3′-UTR of PIK3R1 and AKT3 (Figure 6B), indicating the direct regulation of miR-29b at the 3′-UTR of PIK3R1 and AKT3 transcripts.

To determine whether these findings reflect the regulation of endogenous PIK3R1 and AKT3 by miR-29b, we transiently reintroduced pre-miR-29b into two HSC lines LX1 and HSC-T6. Ectopic expression of miR-29b remarkably reduced protein expression of PIK3R1, AKT3 and phosphorylated AKT (p-AKT) in both cell lines (Figure 6C), suggesting that PIK3R1 and AKT3 are bona fide targets of miR-29b.

### miR-29b inhibits PIK3R1 and AKT3 in liver fibrosis in mice

The effect of miR-29b on protein expression of PIK3R1, AKT3 and p-AKT was examined *in vivo* by Western blot. As shown in Figure 6D, protein expression of PIK3R1, total AKT3 and p-AKT was significantly enhanced in liver fibrotic tissues in CCl_4_-treated mice. In contrast, CCl_4_-treated mice supplemented with miR-29b showed significant reduced expression of PIK3R1, total AKT3 and p-AKT, which paralleled the improvement in histological severity of liver fibrosis (Figure 3A). These results supported the specific inhibitory effect of miR-29b on PIK3R1 expression and AKT3 activation in liver fibrosis.

### Knockdown of PIK3R1 or AKT3 inhibits α-SMA and collagen I

**Figure 7:**
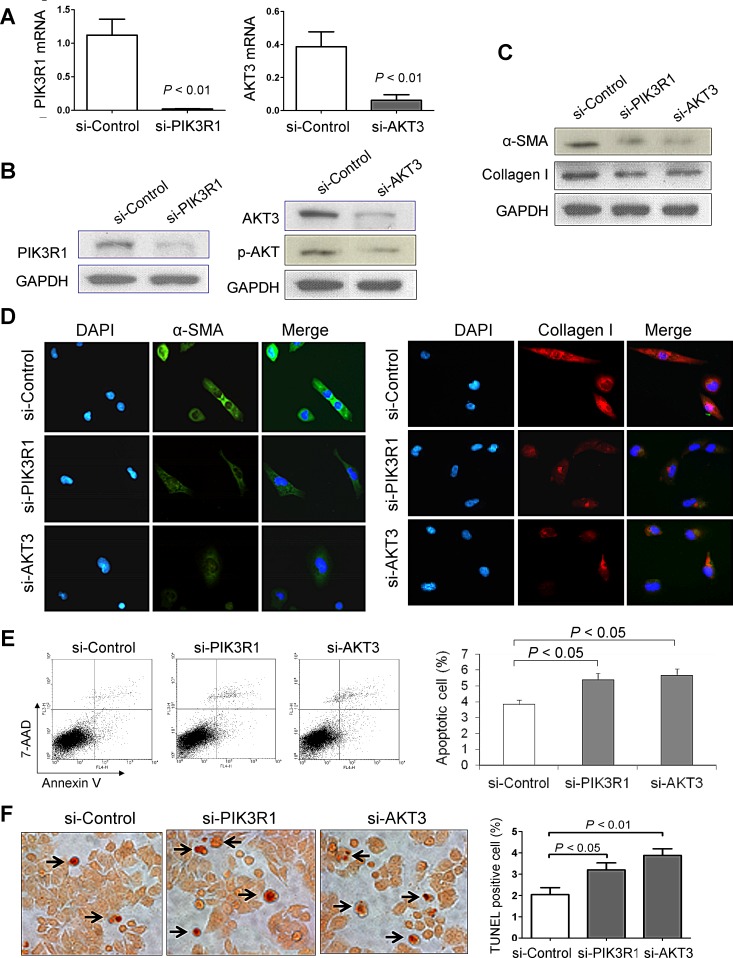
Knockdown of PIK3R1 or AKT3 reduces HSC activation and induces HSC apoptosis (A) Knockdown efficiency of PIK3R1 or AKT3 in LX1 cell line was confirmed by RT-PCR and (B) by Western blot. (C) Knockdown of PIK3R1 or AKT3 inhibits the protein levels of α-SMA and collagen I in LX1 cells by western blot, and (D) by dual-immunofluorescence staining. (E) Knockdown of PIK3R1 or AKT3 induced Annexin V positive apoptotic LX1 cells by flow cytometry. Values are mean ± SD from four independent experiments in duplicate. (F) Induced apoptosis in LX1 cells by Knockdown of PIK3R1 or AKT3 was confirmed by TUNEL staining. Apoptosis index was quantified by counting the proportion of TUNEL-positive cells.

To characterize the effect of PIK3R1 or AKT3 on liver fibrosis, we knockdown PIK3R1 or AKT3 expression in LX1 cells by siRNA transfection. Knockdown efficiency was examined by real-time RT-PCR (Figure 7A) and Western blot (Figure 7B). Knockdown of PIK3R1 or AKT3 significantly reduced the protein expression of α-SMA and collagen I by Western blot (Figure 7C), which was further confirmed by immunofluorescence assay (Figure 7D).

### Knockdown of PIK3R1 or AKT3 induces HSC apoptosis

We also examined whether knockdown of PIK3R1 or AKT3 influences HSC apoptosis. Flow cytometric analysis of apoptotic LX1 cells after stained with Annexin V/7-AAD showed that knockdown of PIK3R1 or AKT3 caused significant increase in the number of apoptotic cells in LX1, respectively (Figure 7E). This effect was further confirmed by TUNEL staining in LX1 cultured slides. Knockdown of PIK3R1 or AKT3 displayed more TUNEL positive cells compared to control, respectively (Figure 7F).

## DISCUSSION

In this study, we found a significant decrease in the expression of miR-29b in human and rodent liver fibrotic tissues compared to normal liver tissues. Similar down-regulation of miR-29b expression was also observed in activated primary HSCs isolated from the liver of rodent compared to their quiescent phenotype. Downregulation of miR-29 members has been reported to be implicated in various fibrotic diseases including cardiac fibrosis [17], lung fibrosis [18] and liver fibrosis [19]. These data suggested that miR-29b may play a role in liver fibrogenesis and its repression may be associated with HSCs activation. The mechanism of miR-29b downregulation in liver fibrosis is therefore evaluated. We found that TGF-β1 down-regulates miR-29b in HSC cells, which was associated with a marked upregulation of p-Smad3 and α-SMA. The signaling mechanism through which TGF-β1 regulates miR-29b expression was examined and we revealed that TGF-β1 downregulates miR-29b expression through the mechanism of Smad3. The interaction of Smad3 with miR-29b came from the findings that the conserved Smad3-binding site was found in the promoter region of miR-29b and that Smad3 was able to interact with the miR-29b promoter region as detected by the ChIP-PCR assay (Figure 1G). Thus, miR-29b is a downstream target gene of Smad3 in liver fibrosis that is negatively regulated by TGF-β/Smad3 signaling. These findings are consistent with our recent reports in pulmonary fibrosis [16] and in renal fibrosis [15] that Smad3 mediates TGF-β1-induced downregulation of miR-29b by binding to miR-29b promoter.

If miR-29b plays a key part in liver fibrogenesis, it would be important to establish that its overexpression ameliorated severity of liver fibrosis. To test this, we used an ultrasound-microbubble-mediated gene transfer to introduce miR-29b into the liver in mice treated with CCl_4._ We have previously shown that the use of ultrasound-microbubble-mediated gene transfer is able to effectively deliver Dox-inducible miR-29b plasmid into kidney to block activation of TGF-β/Smad signaling, thereby inhibiting progressive renal fibrosis in rat model [15]. In this study, we effectively delivered miR-29b plasmid into both normal and disease liver in which higher levels of miR-29b transgene were expressed as detected by *in situ* hybridization and by real-time PCR without any side effect (Figure 2). In the eight-week CCl_4_-induced liver fibrosis model, transduction of miR-29b significantly repressed the severity of hepatic fibrosis as evidenced by reduced collagen deposition and collagen content (Figure 3A-3C). We showed that expression of α-SMA, collagen I, and TIMP-1 were upregulated in CCl_4_-treated mice. These genes are mainly producing by HSCs during fibrogenesis [20]. Introduction of miR-29b resulted in significant downregulation of α-SMA, collagen I and TIMP-1 expression, which is more likely the result of reduced activation of HSCs. In addition, transduction of miR-29b was able to inhibit the activation of Smad3 both *in vitro* and *in vivo*, an important player in fibrogenic pathway, which indicated that miR-29b is not only a downstream target of TGF-β/Smad3 in liver fibrogenesis, but also a negative feedback-regulator of the TGF-β/Smad3 signaling axis in the pathogenesis of liver fibrosis. Therefore, the antifibrotic effect of miR-29b *in vivo* is at least in part due to a decreased accumulation of activated matrix producing HSCs, reduced collagen production as well as increased matrix degradation, thereby blocking fibrosis development (Figure 3E). To clarify this hypothesis, an *in vitro* study on cultured HSCs is warranted to determine the direct effect by which miR-29b protects against fibrosis.

To further define the effect of endogenous transactivation of miR-29b and to understand the functional consequences and molecular basis in liver fibrosis, we examined its direct regulation of HSC biology, a principal mechanism implicated in the antifibrotic effect of miR-29b. We transduced pre-miR-29b in activated human (LX-1) and rat (T6) HSCs. In keeping with the findings in mice, ectopic expression of miR-29b inhibited the expression of α-SMA, collagen I, TIMP-1 and p-Smad3 in both HSC cell lines, suggesting that miR-29b negatively regulated fibrosis by targeting the process of collagen matrix synthesis through inhibiting the activation of HSCs. Moreover, ectopic expression of miR-29b caused a growth arrest in both LX-1 and T6 HSCs as evidenced by cell viability and colony formation assays. To further investigate the mechanism by which miR-29b regulates cell growth, we performed FACS; cell cycle distribution analysis revealed significantly more miR-29b transfected HSCs were arrested in the G1 phase, with a concomitant reduction in cellular proliferation compared with miR-control transfected HSCs. Cell cycle arrest caused by the overexpression of miR-29b was associated with induction of cyclin D1 and p21^cip1^ (Figure 4). These findings imply that miR-29b reduces liver fibrosis by mechanisms of reducing the number of HSCs via causing cell cycle arrest and suppressing cell proliferation.

When liver fibrosis resolves, the activated HSCs are either returning to a quiescent state or undergoing apoptosis [9,21], which causes a decrease in the number of activated HSC. The induction of apoptosis in HSCs by miR-29b was also observed concomitantly with the inhibition of cellular proliferation, whereby apoptosis was executed by the regulation of casepase-9 and PARP (Figure 5). Increased expression of miR-29b activated caspase-9, triggering the proteolytic cleavage of the PARP leading to cellular disassembly and apoptosis. Lots of attention has focused on the process of activated HSC apoptosis because stimulation of this process *in vivo* promotes accelerated rates of the resolution of liver fibrosis [13]. Here we showed that transfection of miR-29b could significantly increase the susceptibility of HSCs to caspase-mediated apoptosis, indicating that apoptosis is an additional mechanism of anti-fibrotic effect of miR-29b in HSCs [22]. Collectively, our *in vitro* findings served as a direct evidence for the regulatory role of miR-29b in HSC activation.

Having shown that miR-29b is a crucial mediator in repressing liver fibrosis through suppressing the activation of HSCs, we looked for the possible downstream effectors participating in its function. Of note, a single miRNA can regulate a multitude of target genes concomitantly. It has been reported that miR-29b suppresses progression of renal fibrosis by down-regulating tropomyosin 1 and COL2A1 [23]. Among the miRNAs predicted to target genes, we revealed for the first time that PIK3R1 and AKT3 act as critical effectors of miR-29b in liver fibrosis. We confirmed the direct interaction of miR-29b in negatively regulating PIK3R1 and AKT3 at their 3′-UTR regions in HSC cells by luciferase activity assay (Figure 6A-6B). Moreover, miR-29b dramatically decreased the protein expression of PIK3R1, AKT3 and p-AKT3 in HSC cells and in fibrotic animal models, indicating the translational repression of PIK3R1 and AKT3 by miR-29b (Figure 6C-6D). Knockdown of PIK3R1 or AKT3 in HSCs led to inhibition of α-SMA and collagen I (Figure 7) and induction of apoptosis. PIK3R1 is a regulatory subunit of phosphatidylinositol 3-kinase (PI3K). Activation of PI3K is important for HSC proliferation *in vitro* and for hepatic fibrogenesis in rodent induced by CCl_4_ [24,25]. PI3K activation leads to the activation of its key downstream kinase, AKT, which stimulates cell proliferation and inhibits apoptosis in HSCs [26]. The PI3K/Akt signaling pathway plays important roles in the development and progression of haptic fibrosis through regulating extracellular matrix degradation and HSC activation [27]. These findings suggest a possible mechanism by which miR-29b suppresses liver fibrosis through negatively regulates PI3K/AKT signaling pathway via direct interaction with PIK3R1 and AKT3. This information highlights the potential therapeutic mechanism and benefit of miR-29b in inhibiting the PI3K/AKT pathway to prevent and treat liver fibrosis.

In conclusion, miR-29b was downregulated in liver fibrosis and was negatively regulated by Smad3 *in vivo* and in HSC cells. Importantly, miR-29b was an antifibrotic factor and ultrasound-microbubble-mediated miR-29b tranduction has prodigious therapeutic potential for liver fibrosis by inhibition of collagen production, stimulation of matrix degradation and repression the activation of HSCs. The molecular mechanisms by which miR-29b exerted its antifibrotic function was by directly inhibiting PIK3R1 and AKT3, causing inactivation of the PI3K/AKT signaling pathway, and ultimately inducting apoptosis of activated HSCs.

## MATERIALS AND METHODS

### Patients and tissues

Liver biopsy tissues were obtained from 20 patients with liver fibrosis and 13 healthy subjects from Prince of Wales Hospital, The Chinese University of Hong Kong, and Queen Marry Hospital, The University of Hong Kong, Hong Kong. Written consent was obtained prior to sample collection, and the study was approved by the Human Ethics Committee of The University of Hong Kong and The Chinese University of Hong Kong. Tissue biopsies were immediately snap-frozen in liquid nitrogen and stored at −80°C for later analysis.

### Animal models of liver fibrosis

Liver fibrosis was induced by intraperitoneal (i.p.) administration of CCl_4_ (1:1 in olive oil) at a dose of 0.2 ml/100 g body weight twice-weekly [28,29]. Control animals were treated by i.p. injection of olive oil only. The animals were sacrificed under anesthesia after 8 weeks of CCl_4_ treatment. The liver tissues were either snap frozen in liquid nitrogen and stored at −80°C or fixed in 10% buffered formalin overnight before embedded in paraffin in preparing for histopathological and immunohistochemical examinations. Experiments were conducted in accordance with principles outlined in the Animal Experimentation Ethics Committee Guide for the Care and Use of Laboratory Animals, and were approved by the Animal Experimentation Ethics Committee of the Chinese University of Hong Kong.

### Ultrasound-mediated miR-29b transfer in the liver

A mixed solution that contained pTRE2-miR-29b and Tet-on plasmids/Sonovue (Bracco Diagnostics, Princeton, NJ) in the ratio of 1:1 (vol:vol) or the control empty vectors (pTRE2-Tet-on/Sonovue) in 200 μl [15], was co-transfected into the liver through tail vein injection followed by 5 min ultrasound treatment (2W/cm2) transcutaneously at liver location (THERASONIC 450, Electro-Medical Supplies, Greenham, England). To induce miR-29b transgene expression, Doxycycline hyclate (Sigma-Aldrich, St. Louis, MO) was supplied in drinking water with a concentration of 200ug/ml during the expeiments. As high expression level of miR-29b was demonstrated to last for 3 weeks after a single dose injection in the pilot study, tail vein injection was performed at about 2 and half weeks for 3 times in total in 8 weeks duration. The numbers of mice in the different experimental groups were: Olive oil-treated group, 6; CCl_4_-treated group, 8; CCl_4_ and empty vector-treated group, 8; and, CCl_4_ and pTRE_2_-miR-29b-treated group, 8.

### ChIP assay

ChIP assay was performed using Transcription Factor ChIP kit (Diagenode, Liège, Belgium). LX1 cells pretreated with TGF-β1 (10 ng/ml) were cross-linked with 1% formaldehyde for 10 min at 37°C. The crosslinking reaction was stopped by adding glycine and collected for nuclei protein extraction. Chromatins from extracted crosslinked nuclei were sheared by sonication using a Bioruptor (Diagenode, Liège, Belgium) to generate 200- to 300-bp DNA fragments, and then precipitated with antibody for Smad3 (Upstate, Billerica, MA), followed by capturing immunoprecipitated proteins/DNA complex with protein-G-magnetic beads. The same amount of non-specific IgG was used as control. Precipitated DNAs were amplified by PCR using specific primers of the promoter of miR-29b with Smad3 binding motif: 5′-TGCTTGGAAAGGTGAGGATG-3′ and 5′-CCCAACCAAAGGCGGACAGC-3′. The fold enrichment was calculated relative to the background detected with non-specific IgG using the following formula: fold enrichment = 2^ (CT of antibody − CT of IgG).

### Dual-luciferase reporter activity assay

The potential miR-29b binding targets were predicted by TargetScan (www.targetscan.org) and miRanda (www.microRNA.org). Sequence of segments with the wild-type or mutant seed region of PIK3R1, AKT3, collagen type III alpha 1 (Col3A1) and collagen type I alpha 2 (Col1A2) were synthesized and cloned into pMIR-REPORT luciferase vector (Ambion, Inc., Grand Island, NY) between Hindlll and Spel restriction sites. The synthesized oligos were shown in Supplementary Table 1. All constructs were verified by sequencing. LX-1 cells (1×105 cells/well) transiently transfected with pre-miR-29b (20 nM) or miR-control (20 nM) were seeded in 24-well plates. pMIR-REPORT vector (195 ng/well) and pRL-TK vector (5 ng/well) were cotransfected using lipofectamine 2000 (Invitrogen). Cells were harvested 48 hours post-transfection and luciferase activities were analyzed by the dual-luciferase reporter assay system (Promega, Madison, WI).

### Immunohistochemistry

Immunohistochemistry was performed on paraffin-embedded liver sections of human normal tissues and fibrosis tissues using the specific antibody for PIK3R1 (Origene Technologies) or AKT3 (Santa Cruz Biotechnology, Santa, Cruz, CA). Negative control was replacement of primary antibody with non-immune serum. The sections were then incubated with biotinylated secondary antibodies (1:400) and the avidin-biotin complex (Histostain - Plus Kits, invitrogen, MD), developed with diaminobenzidine tetrahydrochloride substrate for 3 min and counterstained with hematoxylin.

### Dual-fluorescent immunohistochemistry

Cells grown on sterile slides in 6 well plates were fixed with 3% paraformaldehyde and permeated with 0.1% Triton X-100. Nonspecific binding was blocked with 1% BSA for 30 minutes. The cells were then incubated with primary antibodies against PIK3R1 (OriGene, Rockville, MD), AKT3 (Santa Cruz), phosphorylated-AKT (Cell Signaling, Beverly, MA), α-SMA (Millipore, Billerica, MA) and collagen I (SouthernBiotech, Birmingham, AL) for 1 hour, followed by secondary antibodies for 30 minutes. Nuclei were counterstained by 4′,6-diamidino-2-phenylindole (DAPI) (Invitrogen). Images were captured using confocal microscope (Nikon Eclipse TE2000-S).

### RNA interference and transfection

Human LX1 cells were transfected with siRNA against PIK3R1 or siRNA against AKT3, and control siRNA-A (Santa Cruze) consisting of a scrambled sequence that will not lead to specific degradation of any cellular message. The expression vectors were transfected into LX1 cells using lipofectamine 2000 (Invitrogen). Knockdown efficiency was evaluated by real-time RT-PCR and Western blot.

### Picrosirius red staining for liver collagen and image analysis of liver fibrosis

Liver paraffin sections of 5μm thick were processed with 0.1% Picrosirius red for collagen visualization. Morphometric analysis was then carried out on a computerized image analysis system (Axiocam, Carl Zeiss Microscopy, Oberkochen, Germany) and Bioquant Nova Prime software (Bioquant Image Analysis Corporation, TN). The entire liver section on a slide was capture by consecutive fields at a magnification of x100 without overlapping. The mean of red color stained area of all fields in each section was calculated. The mean area of fibrosis in μm^2^ per field was calculated for each liver section [29].

### Hydroxyproline analysis for collagen content

To quantify collagen content, hepatic hydroxyproline assay was performed (Jiancheng Bioengineering, Nanjing, China). Around 80 mg of liver tissues were homogenized in ice-cold buffer. The homogenates were incubated overnight at 37°C in 5% KOH. The hydrolysates were dried by speed vacuum centrifugation over 3~5 h and redissolved in a buffer solution. Hydroxyproline levels in the hydrolysates were measured at 550 nm. A standard curve of samples with known quantities of hydroxyproline was generated for each assay [29]. Each sample was assayed in triplicate.

### HSC culture

Immortalized human HSC cell line (LX-1) (a generous gift of Prof. SL Friedman of the Mount Sinai School of Medicine, NY) and immortalized rat HSC cell line (HSC-T6) (ATCC, Manassas, VA) were used for the *in vitro* experiments. Both LX-1 and HSC-T6 exhibit morphologic and functional characteristics of activated HSCs. For some experiments, we used primary HSCs isolated from male Sprague-Dawley rats. HSCs are prepared by collagenase/pronase digestion of rat liver using a perfusion system and subsequent fractionation of the heterogeneous cell suspension on continuous density gradients. Stellate cells are the least dense fraction of the nonparenchymal cells, and they float effectively away from other hepatic cells during centrifugation. When cultured on plastic, freshly isolated hepatic stellate cells undergo spontaneous activation. The HSCs maintain quiescent at day 2 of culture, and will be fully activated at day 7 in culture. The cells were maintained in Dulbecco's modified Eagle's medium (DMEM, GIBCO, Grand Island, NY) supplemented with 10% fetal bovine serum.

### miR-29b transfection in HSCs

miR-29b precursor (pre-miR-29b) and the miRNA Mimic Negative Control (pre-miR-control) (Ambion Life Technologies, Austin, TX) were transiently transfected into HSCs (LX-1, HST-T6) using Lipofectamine 2000 (Invitrogen, Carlsbad, CA) for 48 h and 72 h, respectively. Total RNA and protein were isolated from the cell pellets to evaluate the transfection efficiency of miR-29b in HSCs.

### RNA extraction and PCR analyses

Total RNA was extracted from cell pellets or tissues using QIAzol reagent (Qiagen, Hilden, Germany). Semi-quantitative RT-PCR was performed using Hot-start DNA polymerase (Invitrogen) with the housekeeping gene β-actin as an internal control. Quantitative real-time PCR was performed using SYBR Green master mixture on HT7900 system (Applied Biosystems, Foster City, CA). The expression level of mature miR-29b was quantified by TaqMan microRNA assays (Applied Biosystems). The comparative ΔCt method was used to calculate the relative abundance of miRNA compared with RNU6B expression (Fold difference relative to RNU6B).

### Western blot

Total protein was extracted from cells pellet. Thirty micrograms of protein from each sample were separated on 12% SDS-PAGE and transferred onto nitrocellulose membranes (GE Healthcare, Piscataway, NJ). Blots were immunostained with primary antibody and secondary antibody, respectively, with GAPDH served as a loading control.

### Cell viability assay

After transfection, the LX-1 and HSC-T6 cells were digested and re-seeded in 96-well plates (3×10^3^ per well) for cell viability assay using the (3-(4,5-dimethylthiazol-2-yl)-5-(3-carboxymethoxyphenyl)-2-(4-sulfophenyl)-2H-tetrazolium, inner salt) (MTS) assay (Promega, Madison, WI). Twenty uL of reaction solution was added to cultured cells in 100 uL culture medium and incubated at 37°C for 1.5 h. The optical density was measured at a wavelength of 490 nm. The cell viability assay was carried out in four wells for three independent experiments.

### Colony formation assay

LX-1 and HSC-T6 cells (5 × 10^4^/well) were plated in a 24-well plate and transfected with pre-miR-29b or control RNA. After 48 h of transfection, cells were collected and seeded (2×10^3^/well) in 6-well plate for 10 to 14 days. Colonies (≥ 50 cells/colony) were then counted after being fixed with 70% ethanol and stained with 5% Gentian Violet (ICM Pharma, Singapore, Singapore).

### Cell cycle analysis

LX1 cells transfected with pre-miR-29b or miR-control were fixed in 70% ethanol-PBS for 24 hours. Cellular DNA was stained with 50 μg/ml propidium iodide (BD Pharmingen, Franklin Lakes, NJ), and then sorted by FACSCalibur (BD Biosciences, Bedford, MA). Cell-cycle profiles were analyzed by ModFit 3.0 software (Becton Dickinson, San Diego, CA).

### Apoptosis assays

Apoptosis was determined by dual staining with APC Annexin V (BD Biosciences) and 7-amino-actinomycin (7-AAD) (BD Biosciences) with subsequent flow cytometry analysis. The relative proportion of Annexin V-positive and 7-AAD negative cells was determined using the ModFitLT software (Becton Dickinson, San Diego, CA) and counted as early apoptotic cells (Annexin V-positive, 7-AAD-negative), late apoptotic cells (Annexin V-positive, 7-AAD-positive), necrotic cells (Annexin V-negative, 7-AAD-positive) and viables (Annexin V-negative, 7-AAD-negative),.

In addition, terminal deoxynucleotidyl TUNEL staining was also employed for detection of apoptosis in liver sections (Promega, Madison, WI). The apoptosis index was calculated as the percentage of TUNEL-positive cells which showed clear brown nuclear staining (n >1000).

### Statistical analysis

Data are presented as mean ± SD. Statistical analysis was performed using an unpaired Student's *t*-test for single, or analysis of variance for multiple, group comparison. A *P* value of less than 0.05 was considered significant.

## SUPPLEMENTARY MATERIAL AND TABLE



## Abbreviations

3′UTR: 3′-untranslated regions
AAD: Aminoactinomycin D
AKT: Protein Kinase B
α-SMA: alpha-smooth muscle actin
CCl4: carbon tetrachloride
cDNA: complementary DNA
DDR2: discoidin domain receptor 2
ChIP: chromatin immunoprecipitation
Col3a1: collagen type III alpha 1
Col1A2: collagen type I alpha 2
DAPI: 4′,6-diamidino-2-phenylindole
ECM: extracellular matrix
FACS: fluorescence-activated cell sorting
FN1: fibronectin 1
HSCs: hepatic stellate cells
i.p.: intraperitoneal
ITGB1: integrin β1
miRNA: microRNA
MMP: matrix metalloproteinases
PDGFR-β: platelet-derived growth factor receptor-β
PIK3R1: phosphoinositide-3-kinase regulatory subunit 1
p-Smad3: phosphorylated-Smad3
RT-PCR: reverse transcription polymerase chain reaction
TGF-β: transforming growth factor-β
TIMP-1: tissue inhibitors of metalloproteinase 1PARP
poly (ADP-ribose): polymerase 1
TUNEL: terminal deoxynucleotidyl transferase-mediated nick-end labeling.

## References

[1] Popov Y, Schuppan D (2009). Targeting Liver Fibrosis. Strategies for Development and Validation of Antifibrotic Therapies. Hepatology.

[2] Albanis E, Friedman SL (2006). Antifibrotic agents for liver disease. Am J Transplant.

[3] Moreira RK (2007). Hepatic stellate cells and liver fibrosis. Arch Pathol Lab Med.

[4] Reeves HL, Friedman SL (2002). Activation of hepatic stellate cells - A key issue in liver fibrosis. Front Biosci.

[5] Dooley S, Delvoux B, Lahme B, Mangasser-Stephan K, Gressner AM (2000). Modulation of transforming growth factor beta response and signaling during transdifferentiation of rat hepatic stellate cells to myofibroblasts. Hepatology.

[6] Galli A, Crabb DW, Ceni E, Salzano R, Mello T, Svegliati-Baroni G, Ridolfi F, Trozzi L, Surrenti C, Casini A (2002). Antidiabetic thiazolidinediones inhibit collagen synthesis and hepatic stellate cell activation *in vivo* and *in vitro*. Gastroenterology.

[7] Iredale JP (2001). Hepatic stellate cell behavior during resolution of liver injury. Semin Liver Dis.

[8] Benyon RC, Arthur MJ (2001). Extracellular matrix degradation and the role of hepatic stellate cells. Semin Liver Dis.

[9] Friedman SL, Bansal MB (2006). Reversal of hepatic fibrosis - Fact or fantasy?. Hepatology..

[10] Bartel DP (2009). MicroRNAs: Target Recognition and Regulatory Functions. Cell.

[11] Ura S, Honda M, Yamashita T, Ueda T, Takatori H, Nishino R, Sunakozaka H, Sakai Y, Horimoto K, Kaneko S (2009). Differential microRNA expression between hepatitis B and hepatitis C leading disease progression to hepatocellular carcinoma. Hepatology.

[12] Jin X, Ye YF, Chen SH, Yu CH, Liu J, Li YM (2009). MicroRNA expression pattern in different stages of nonalcoholic fatty liver disease. Dig Liver Dis.

[13] Murakami Y, Yasuda T, Saigo K, Urashima T, Toyoda H, Okanoue T, Shimotohno K (2006). Comprehensive analysis of microRNA expression patterns in hepatocellular carcinoma and non-tumorous tissues. Oncogene.

[14] Ji J, Zhang J, Huang G, Qian J, Wang X, Mei S (2009). Over-expressed microRNA-27a and 27b influence fat accumulation and cell proliferation during rat hepatic stellate cell activation. FEBS Lett.

[15] Qin W, Chung AC, Huang XR, Meng XM, Hui DS, Yu CM, Sung JJ, Lan HY (2011). TGF-β/Smad3 signaling promotes renal fibrosis by inhibiting miR-29. J Am Soc Nephrol.

[16] Xiao J, Meng XM, Huang XR, Chung AC, Feng YL, Hui DS, Yu CM, Sung JJ, Lan HY (2012). miR-29 inhibits bleomycin-induced pulmonary fibrosis in mice. Mol Ther.

[17] van Rooij E, Sutherland LB, Thatcher JE, DiMaio JM, Naseem RH, Marshall WS, Hill JA, Olson EN (2008). Dysregulation of microRNAs after myocardial infarction reveals a role of miR-29 in cardiac fibrosis. Proc Natl Acad Sci U S A.

[18] Cushing L, Kuang PP, Qian J, Shao F, Wu J, Little F, Thannickal VJ, Cardoso WV, Lü J (2011). miR-29 is a major regulator of genes associated with pulmonary fibrosis. Am J Respir Cell Mol Biol.

[19] Roderburg C, Urban GW, Bettermann K, Vucur M, Zimmermann H, Schmidt S, Janssen J, Koppe C, Knolle P, Castoldi M, Tacke F, Trautwein C, Luedde T (2011). Micro-RNA profiling reveals a role for miR-29 in human and murine liver fibrosis. Hepatology.

[20] Friedman SL (2000). Molecular regulation of hepatic fibrosis, an integrated cellular response to tissue injury. J Biol Chem.

[21] Wright MC, Issa R, Smart DE, Trim N, Murray GI, Primrose JN, Arthur MJ, Iredale JP, Mann DA (2001). Gliotoxin stimulates the apoptosis of human and rat hepatic stellate cells and enhances the resolution of liver fibrosis in rats. Gastroenterology.

[22] Elsharkawy AM, Oakley F, Mann DA (2005). The role and regulation of hepatic stellate cell apoptosis in reversal of liver fibrosis. Apoptosis.

[23] Fang Y, Yu X, Liu Y, Kriegel AJ, Heng Y, Xu X, Liang M, Ding X (2013). miR-29c is downregulated in renal interstitial fibrosis in humans and rats and restored by HIF-α activation. Am J Physiol Renal Physiol.

[24] Reif S, Lang A, Lindquist JN, Yata Y, Gabele E, Scanga A, Brenner DA, Rippe RA (2003). The role of focal adhesion kinase-phosphatidylinositol 3-kinase-Akt signaling in hepatic stellate cell proliferation and type I collagen expression. J Biol Chem.

[25] Gentilini A, Marra F, Gentilini P, Pinzani M (2000). Phosphatidylinositol-3 kinase and extracellular signal-regulated kinase mediate the chemotactic and mitogenic effects of insulin-lice growth factor-I in human hepatic stellate cells. J Hepatol.

[26] Parsons CJ, Takashima M, Rippe RA (2007). Molecular mechanisms of hepatic fibrogenesis. J Gastroenterol Hepatol.

[27] Voloshenyuk TG, Landesman ES, Khoutorova E, Hart AD, Gardner JD (2011). Induction of cardiac fibroblast lysyl oxidase by TGF-β1 requires PI3K/Akt, Smad3, and MAPK signaling. Cytokine.

[28] Yu J, Wu CW, Chu ES, Hui AY, Cheng AS, Go MY, Ching AK, Chui YL, Chan HL, Sung JJ (2008). Elucidation of the role of COX-2 in liver fibrogenesis using transgenic mice. Biochem Biophys Res Commun.

[29] Yu J, Zhang S, Chu ES, Go MY, Lau RH, Zhao J, Wu CW, Tong L, Zhao J, Poon TC, Sung JJ (2010). Peroxisome proliferator-activated receptors gamma reverses hepatic nutritional fibrosis in mice and suppresses activation of hepatic stellate cells *in vitro*. Int J Biochem Cell Biol.

