# Supplementary material for: microRNA-29b prevents liver fibrosis by attenuating hepatic stellate cell activation and inducing apoptosis through targeting PI3K/AKT pathway
Source: Oncotarget. 2014 Oct 22;6(9):7325–38. doi: 10.18632/oncotarget.2621 (PMC4466688; doi:10.18632/oncotarget.2621)
Supplement: Supplementary file 1 [file oncotarget-06-7325-s001.pdf]

microRNA-29b prevents liver fibrosis by attenuating hepatic stellate cell activation and inducing apoptosis through targeting PI3K/AKT pathway

Supplementary Material

S Table 1. Sequence alignments of miR-29b potential binding sites in 3'UTR area of PIK3R1, AKT3, Col1A2 and Col3A1 with wild-type and mutant forms

PIK3R1	Wild type	5'-ctagtaaagaggcctttaaccaTGGTGCTtgttaatgctttctgaagagct-3'<br>5'-cttcagaaagcattaacaAGCACCAtggttaaaggcctcttta-3'
	Mutant	5'-ctagtaaagaggcctataaccaACGCTTGtgttaatgctttctgaagagct-3'<br>5'-cttcagaaagcattaacaCAAGCGTtggttataggcctcttta-3'
AKT3	Wild type	5'-ctagtcagattaaacccttTGGTGCTAggagctgacaatttccaaagagct-3'<br>5'-ctttggaaattgtcagctccTAGCACCAaagggtttaatctga-3'
	Mutant	5'-ctagtcagactaaacccttACGGTTATggagctgacaatttccaaagagct-3'<br>5'-ctttggaaattgtcagctccATAACCGTaagggtttagtctga-3'
Col1A2	Wild type	5'-ctagtgttttgttcataatacaaaGGTGCTAattaatagtatttcaggagct-3'<br>5'-cctgaaatactattaatTAGCACCtttgtattatgaacaaaaca-3'
	Mutant	5'-ctagtgttttgttcataatacaaaTTGTTACattaatagtatttcaggagct-3'<br>5'-cctgaaatactattaatGTAACAAtttgtattatgaacaaaaca-3'
Col3A1	Wild type	5'-ctagtaaaatgtctcaaTGGTGCTAtaataaataaacttcaacactctgagct-3'<br>5'-cagagtgttgaagtttatttattaTAGCACCAttgagacatttta-3'
	Mutant	5'-ctagtaaaatgtctcaaGATCAACCtaataaataaacttcaacactctgagct-3'<br>5'-cagagtgttgaagtttatttattaGGTTGATCttgagacatttta-3'
